# Seabirds in crisis: Plastic ingestion induces proteomic signatures of multiorgan failure and neurodegeneration

**DOI:** 10.1126/sciadv.ads0834

**Published:** 2025-03-12

**Authors:** Alix M. de Jersey, Jennifer L. Lavers, Alexander L. Bond, Richard Wilson, Graeme R. Zosky, Jack Rivers-Auty

**Affiliations:** ^1^ Tasmanian School of Medicine, College of Health and Medicine, University of Tasmania, 17 Liverpool Street, Hobart, Tasmania 7000, Australia.; ^2^ Gulbali Institute, Charles Sturt University, Wagga Wagga, New South Wales 2678, Australia.; ^3^ Esperance Tjaltjraak Native Title Aboriginal Corporation, 11A Shelden Road, Esperance, Western Australia 6450, Australia.; ^4^ Bird Group, The Natural History Museum, Akeman Street, Tring, Hertfordshire HP23 6AP, UK.; ^5^ Central Science Laboratory (CSL), University of Tasmania, Sandy Bay, Tasmania 7005, Australia.; ^6^ Menzies Institute for Medical Research, College of Health and Medicine, University of Tasmania, 17 Liverpool Street, Hobart, Tasmania 7000, Australia.

## Abstract

Understanding plastics’ harmful impacts on wildlife would benefit from the application of hypothesis agnostic testing commonly used in medical research to detect declines in population health. Adopting a data-driven, proteomic approach, we assessed changes in 745 proteins in a free-living nonmodel organism with differing levels of plastic exposure. Seabird chicks heavily affected by plastic ingestion demonstrated a range of negative health consequences: Intracellular components that should not be found in the blood were frequently detected, indicative of cell lysis. Secreted proteins were less abundant, indicating that the stomach, liver, and kidneys are not functioning as normal. Alarmingly, these signatures included evidence of neurodegeneration in <90-day-old seabird chicks with high levels of ingested plastic. The proteomic signatures reflect the effects of plastic distal to the site of exposure (i.e., the stomach). Notably, metrics commonly used to assess condition in wildlife (such as body mass) do not provide an accurate description of health or the impacts of plastic ingestion.

## Teaser

Plastic consumption in seabirds causes blood signatures of widespread organ damage including brain, stomach, kidney, and liver.

## INTRODUCTION

In recent years, our awareness of the health consequences of exposure to plastic for species and ecosystems has increased substantially, with plastic ingestion shown to alter blood chemistry (*1*), fatty acids (*2*), and pollutant profiles in wildlife (*3*), as well as the physiochemical properties of habitats (*4*, *5*). This growing body of knowledge is based on methods that test hypotheses using specific targeted techniques (*6*–*8*), and many are completed under controlled laboratory settings (*9*). However, the complex and often synergistic nature of plastic impacts also means that specific and targeted hypothesis testing may miss the complexity of the interactions between plastics and biological systems (*10*). For example, a handful of studies reported no relationship between ingested plastic and seabird body size (*11*, *12*); however, many of the consequences have been linked to exposure to plastic in more recent years [e.g., tissue damage, inflammation, or organ failure; (*13*)], which were not measured. For short-tailed shearwaters *Ardenna tenuirostris*, Cousin *et al.* (*14*) concluded that ingested plastic did not alter body size (a standard metric), but a more in-depth investigation of this same issue using fatty acid analysis later revealed a clear link with the use of more sensitive measures of bird health and body condition (*2*).

Another limitation is the availability of data on whole-of-body impacts associated with exposure to plastics, which requires detailed investigation of pathology distal from the point of exposure (i.e., the stomach). Shedding of nanoplastic particles (<1 μm) from larger items ingested by animals, combined with exposure to chemical leachates associated with plastics, can cause damage to the kidneys and spleen in sable shearwaters *Ardenna carneipes* [previously flesh-footed shearwater; (*13*, *15*)] as well as the stomach (*16*). These types of pathologies have only rarely and recently been investigated, leading some to mistakenly conclude that plastic has no obvious health consequences and is therefore a “distraction” from other environmental crises (*17*). Many of the conclusions drawn regarding the potential health consequences of plastic ingestion, to date, do not adequately reflect the scope or severity of this pollution issue and must therefore be revisited using more targeted techniques.

In human health and medical research, investigations of complex health issues often adopt a hypothesis agnostic approach as research targeting individual parameters of interest leads to poor outcomes and delayed understanding. For example, studies investigating single gene-disease associations in humans frequently yield inconsistent and nonreproducible data (*18*). To address this, a hypothesis agnostic, or data-driven, approach focusing on genome-wide association studies has been used to undertake screening without any prior predilection for specific genes (*19*). For plastics, we now know that research focused on relatively simplistic metrics does not tell the whole story, and there is a clear need to shift to more comprehensive, data-driven approaches that adequately reflect the urgency and complexity of this diverse pollutant (*20*).

The development of omic technologies (e.g., proteomics and transcriptomics) and bioinformatics has shifted the focus of research from relatively simplistic models (e.g., lethal toxicity testing of individual chemicals) to mechanistic, sublethal exploration of whole suites of chemicals simultaneously (*21*). While few biomarkers of pathologic states have been identified in wildlife [except see (*22*, *23*)], the benefits of nontargeted proteomics have been consistently demonstrated in a medical context (*24*, *25*). Such high-throughput methods have provided powerful diagnostic tools for chemical pollutants, providing much-needed data on the underlying mechanisms of toxicity (*22*). However, this technology has yet to be applied to other pollutants, namely plastics, despite increasing awareness of their threat to health and the environment (*26*).

Here, we use a data-independent acquisition mass spectrometry (DIA-MS)–based proteomic approach to investigate the physiological responses in seabirds to the ingestion of plastic. We compare two different levels of plastic exposure (low and high) of sable shearwaters *A. carneipes* with each group having equivalent body size and no discernible differences in morphology or overall health. To date, plastics research has largely focused on opportunistic sampling of deceased or visibly impaired emaciated animals (*12*, *27*); however, here, we report substantial differences in proteomic signatures in live plastic-exposed seabird chicks before fledgling that otherwise appear healthy. To validate these findings, we have performed quantitative polymerase chain reaction (qPCR) on stomach and liver tissues on necropsied chicks to determine the expression of genes of interest identified through proteomics. Furthermore, we performed organ-specific DIA-MS proteomics on brain tissue, which revealed neuroplasticity, indicating that the brain is responding to an injury. The signatures observed resemble those seen in multiorgan failure and neurodegeneration, demonstrating that plastics in the stomach are inducing nonlocal tissue damage and pathology. We hypothesize that this is through nanoplastic shedding and translocation as well as chemical leachates from micro- and macroplastics within the stomach of the bird.

## RESULTS

**Fig. 1.**
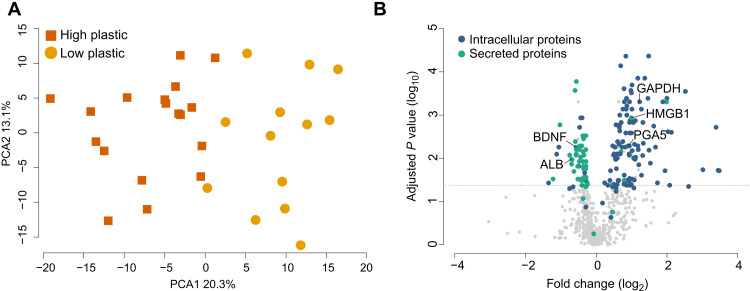
Plastic ingestion induces substantial differences in the plasma proteome. (**A**) Scaled PCA of the proteomic data revealed clear distinction between the proteomes of sable shearwater *A. carneipes* chicks that have ingested low (*n* = 13) or high (*n* = 18) quantities of plastic. (**B**) The volcano plot shows the result of *t* test analysis of the proteomic data and highlights the changes in relative abundance of secreted and intracellular proteins in seabird chicks heavily affected by plastic ingestion. The line indicates the threshold for significance (FDR-adjusted *P* < 0.05), and the proteins of interest that are labeled are BDNF, ALB, GAPDH, HMGB1, and PGA5.

Despite the visual cues suggesting that shearwater chick health was comparable between groups (Table 1), 202 of the 745 (27%) detected plasma proteins differed significantly between low and highly plastic-exposed groups, indicating that substantial physiological changes have occurred because of the presence of plastics. Accordingly, scaled principal component analysis (PCA) revealed a clear separation in the proteome profiles between sable shearwater chicks with low and high quantities of ingested plastic (Fig. 1A).

**Table 1.** Comparisons in morphometric data of live sable shearwater *A. carneipes* chicks exposed to low (*n* = 13) or high (*n* = 18) quantities of ingested plastics sampled for plasma proteomics. Count data are displayed as the median with interquartile ranges (IQR). Continuous data are displayed as the means ± SD. The Poisson generalized linear model for count data and general linear models were used for continuous variables (corrections or transformations were applied where necessary). Comparisons were adjusted using Holm-Šídák post hoc corrections. NS, not significant; ***P < 0.001.

	Low plastic	High plastic	Statistical test
Weight (g)	660 ± 50	670 ± 90	*t*_27.7_ = −0.34, NS
Wing length (mm)	305 ± 9	303 ± 10	*t*_27.5_ = 0.65, NS
Culmen length (mm)	39.8 ± 1.5	38.8 ± 1.5	*t*_26.0_ = 1.72, NS
Head + bill length (mm)	93.2 ± 2.9	91.8 ± 2.5	*t*_23.5_ = 1.45, NS
Ingested plastic count	1 (IQR, 0–5)	27 (IQR, 4–403)	*x*^2^_1_ = 1118.3, ***
Ingested plastic mass (g)	0.03 ± 0.09	6.71 ± 10.12	*R*^2^ = 0.849, *F*_1,29_ = 163.4, ***

### Proteome differences in response to plastic exposure

We used the Protein Atlas database to map the abundance patterns of the 202 significantly different proteins to determine whether they are intracellular proteins or secreted proteins. Most proteins detected at higher abundance in the highly plastic-exposed group were classified as intracellular. This suggests that greater cell lysis had occurred within chicks heavily affected by plastic ingestion (Fig. 1B), resulting in the release of intracellular cellular components into the blood. This is particularly evident in the increase in abundance of intracellular proteins including glyceraldehyde-3-phosphate dehydrogenase (GAPDH), lactate dehydrogenase, and high mobility group box 1 (HMGB1) detected within the plasma as these proteins are commonly used as a specific marker of cell lysis within controlled cell culture experiments (*28*). In contrast, most proteins that were less abundant or not present in the high plastic ingestion group were secreted proteins, indicating a loss or substantial change in organ function, demonstrating the impacts of plastic ingestion on seabird chicks (Fig. 1B).

### Proteome enrichment and organ mapping

**Fig. 2.**
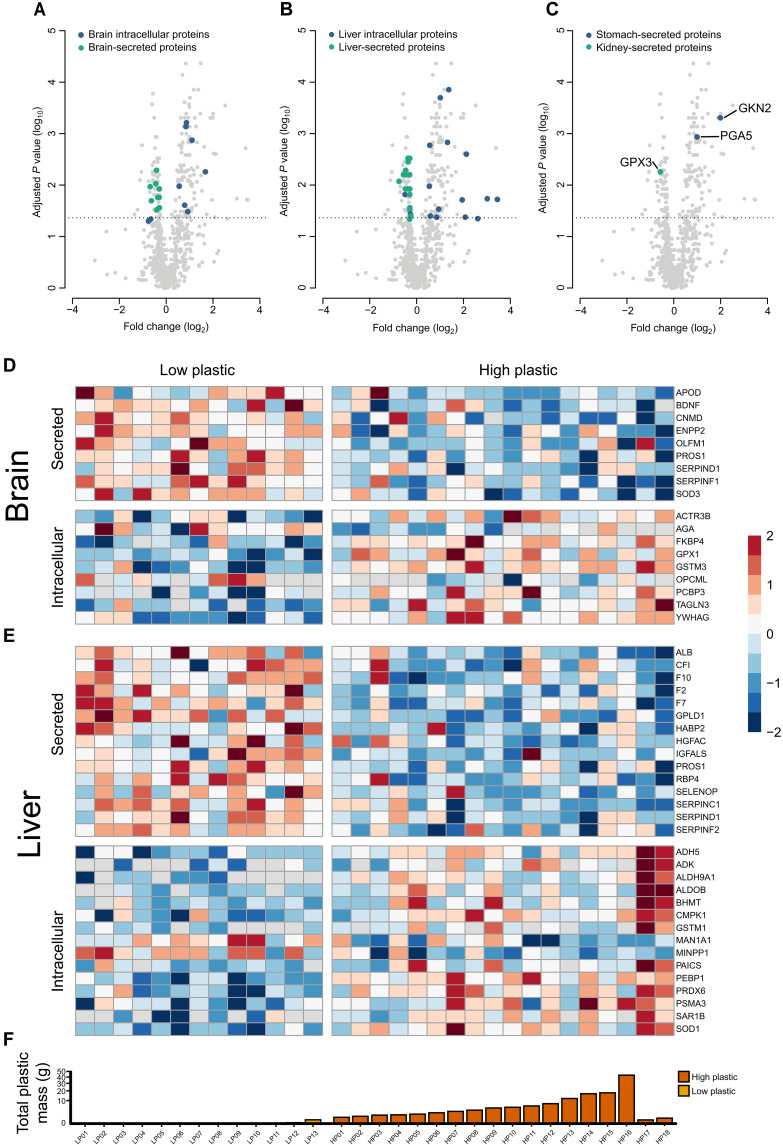
Proteomics demonstrate that plastics cause a decrease in organ-secreted proteins and an increase in intracellular proteins in the plasma of sable shearwater chicks (*A. carneipes*). (**A** to **C**) Volcano plots display the log fold change in protein abundance between chicks with low (*n* = 13) and high (*n* = 18) levels of ingested plastic, indicating that many of the intracellular and secreted proteins from the (A) brain, (B) liver, and (C) kidney and stomach are either more or less relatively abundant between the groups. *t* tests were used to determine significance with the dashed line denoting the significant threshold (FRD-adjusted *P* < 0.05). Proteins of interest include GKN2, GPX3, and PGA5. (**D** and **E**) Heatmaps displaying the standardized relative abundance of secreted and intracellular proteins in the (D) brain and (E) liver between low and high levels of plastic ingestion. Protein abundance was normalized by calculating *Z*-scores. Columns of the heatmaps correspond to individual chicks. (**F**) Total mass of ingested plastic for each chick corresponding to the heatmap, with scale square root transformation applied.

Volcano plots indicate that numerous intracellular and secreted proteins from various organs, including the brain (Fig. 2A), liver (Fig. 2B), stomach, and kidney (Fig. 2C), exhibit altered relative abundance in response to plastic exposure. We performed a nontraditional enrichment analysis to determine patterns in altered biological functions.

Despite the unique property of the proventriculus (hereafter referred to as the stomach) of not releasing secreted proteins into the blood, an increase in stomach-synthesized proteins was observed in the plasma of seabird chicks with high quantities of ingested plastic (Fig. 2). Proteins such as pepsinogen A5 (PGA5) (*29*) and gastrokine-2 (GKN2) (*30*), synthesized in the stomach lining, were found enriched, demonstrating a breakdown in the integrity of the stomach’s permeability. Consequently, proteins typically only found within the stomach are being released into the bloodstream.

Furthermore, in seabird chicks with high quantities of ingested plastic, we saw reduced levels of many critical blood proteins predominantly produced by the liver including albumin, complement proteins, and coagulation proteins (Fig. 2). Conversely, intracellular liver proteins were increased in the plasma, including proteins such as alcohol dehydrogenase and aldehyde dehydrogenase (Fig. 2). This suggests that uncontrolled cell death through necrosis is occurring within the liver.

To determine whether the stomach and liver proteomic results observed are due to a change in protein expression in response to organ damage or loss of integrity in the barriers of the organ-specific vasculature, we performed qPCR to determine the expression of genes of interest between beach-washed seabird chicks with low and high quantities of ingested plastic. Typical proteomic validation approaches, such as an enzyme-linked immunosorbent assay or Western blot, were not possible because of the lack of established antibodies and other tools in seabird species.

**Fig. 3.**
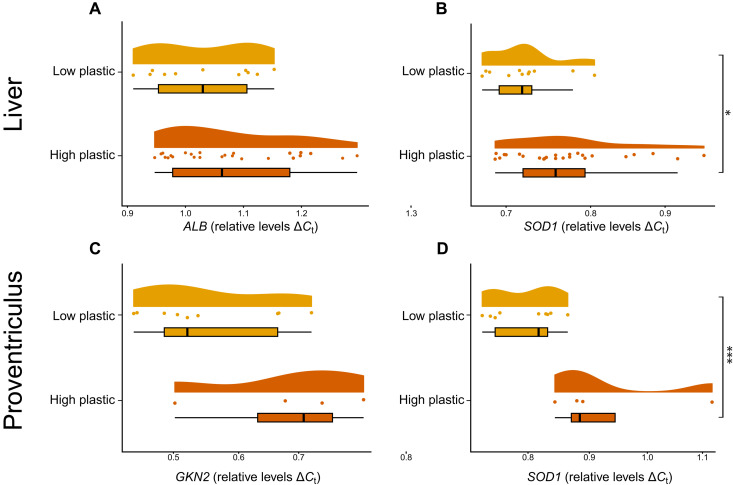
Plastic ingestion induces changes to gene regulation. qPCR on RNA extracted from the (**A** and **B**) liver (*n* = 36) and (**C** and **D**) proventriculus (*n* = 13) of euthanized sable shearwater (*A. carneipes*) chicks with low and high levels of plastic exposure reveals up-regulation of antioxidant pathways. Genes of interest include albumin (*ALB*), superoxide dismutase (*SOD1*), and gastrokine-2 (*GKN2*). Relative *C*_t_ values of target genes of interest were calculated using the delta-delta (ΔΔ*C*_t_) method against the reference housekeeping gene. Hmbs was selected as the housekeeping gene. Gene expression differences between low and high plastic ingestion rates were analyzed with general linear models. Data were Box-Cox transformed and tested for equal variance. *P < 0.05 and ***P < 0.001.

We found no decrease in the gene expression of albumin (*ALB*) or *GKN2* using qPCR (Fig. 3). This supports the interpretations that the up-regulation of organ-specific proteins in the plasma is due to organ leakage rather than organ damage. For example, GKN2 is a protein expressed by the gastric mucosal cells, and paracrine is released into the lumen and acts on surrounding cells. The increase in plasma GKN2 is likely due to the increased permeability of the gut as it additionally coincided with an increase in digestive enzyme PGA5 in the plasma.

Extracellular glutathione peroxidase 3 (GPX3) detectable in the plasma is predominantly produced by the kidneys (*31*). The decrease in abundance of this protein observed is indicative of chronic renal failure (*32*) or chronic kidney disease (*33*) (Fig. 2). Highly plastic-exposed birds showed a significant reduction in the plasma levels of GPX3 (*P* = 0.006).

Concerningly, protein signatures highly associated with neurodegenerative diseases were detected in chicks heavily affected by plastic ingestion (Table 2 and table S1). Of note is brain-derived neurotrophic factor (BDNF), which plays a critical role in the growth, survival, and function of neurons (*34*). The relative level of BDNF was significantly declined in chicks with high plastic ingestion (*P* = 0.01), demonstrating a reduction in the health and function of the brain and nervous system of this group.

**Table 2.** Proteomic analysis reveals that plastic ingestion is associated with signatures of cell lysis, neurodegeneration, response to infection, and mechanisms for damage mitigation and repair. A total of 745 proteins identified in the plasma of sable shearwater (*A. carneipes*) chicks exposed to varying levels of ingested plastic (low *n* = 13 and high *n* = 18) was mapped to function, pathway, or location using Gene Ontology (GO), Kyoto Encyclopedia of Genes and Genomes (KEGG), and WikiPathways databases to determine enrichment. Proteins were evaluated for significant differences between groups using Mann-Whitney tests, with FDR correction applied. Proteins that significantly differed between groups were then analyzed for enrichment location through permutation tests with 10,000 resampling events. All enriched outputs showed increased abundance in the high plastic group. *P < 0.05, **P < 0.01, and ***P < 0.001.

	Enriched pathway	Gene enrichment fraction (plastic, control)
Ubiquitously expressed intracellular proteins	Nucleus (GO: 0005634)	61/151 ***
	Intracellular membrane-bound organelles (GO: 0043231)	66/175 ***
	Cytoskeleton (GO: 0005856)	23/47 ***
	Cadherin binding (GO: 0045296)	21/46 **
	Oxidoreductase activity (GO: 0016616)	6/9 *
	Pathways of neurodegeneration (KEGG)	15/28 **
Neurodegeneration	Huntington disease (KEGG)	12/20 **
	Alzheimer disease (KEGG)	10/20 *
	Amyotrophic lateral sclerosis (KEGG)	13/29 *
	VEGFA-VEGFR2 signaling pathway WP3888	22/52 **
Damage mitigation and repair	NRF2 pathway WP2884	9/14 **

**Fig. 4.**
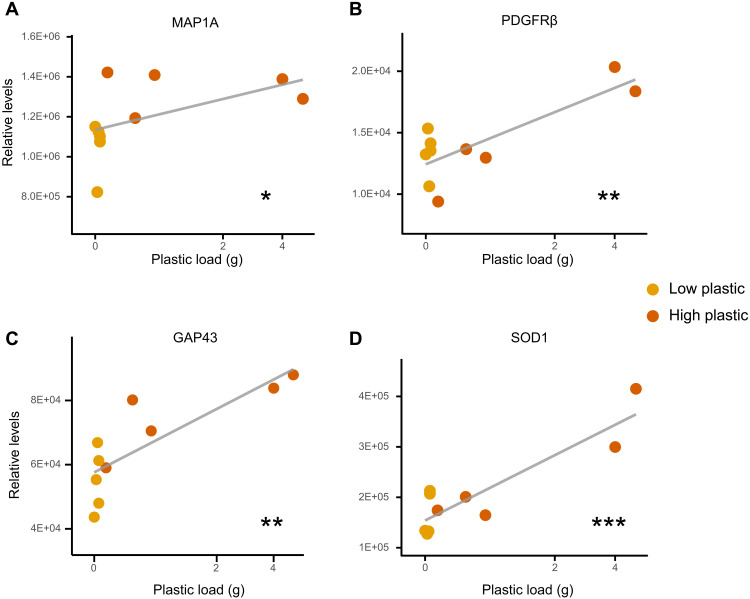
Proteomic analysis on hippocampal brain tissue (*n* = 10) from sable shearwater (*A. carneipes*) chicks reveals a positive correlation between neuroplasticity-associated pathway enrichment and high levels of ingested plastic. Proteins of interest include (**A**) MAP1A, (**B**) PDGFRβ, (**C**) GAP43, and (**D**) SOD1. Linear models were used to assess a dose-dependent relationship between protein relative abundance and ingested plastic load (g), following Box-Cox transformation and testing for homogeneity of variance to ensure that model assumptions were met. *P < 0.05, **P < 0.01, and ***P < 0.001.

Further investigation of plasma markers for poor brain health was performed using proteomic analysis of hippocampal brain tissue of euthanized sable shearwater chicks. Of the 4429 proteins detected, 320 were significantly different between chicks with low and high quantities of ingested plastic (*n* = 5 per group). There was enrichment of neurogenesis pathways and tubulin (table S2; *P* = 0.098 and *P* = 0.058). Both neurogenesis (Fig. 4C) (*35*, *36*) and microtubule assembly (Fig. 4A) (*37*) are markers of brain injury, which is being responded to by the development of fibrosis [platelet-derived growth factor receptor β (PDGFRβ); Fig. 4B] (*38*) and an adaptive cellular response to the increase in oxidative stress [superoxide dismutase 1 (SOD1); Fig. 4D] (*39*).

## DISCUSSION

Despite the absence of visible signs of ingesting high quantities of plastic, this study revealed significant differences in the proteomes of free-living sable shearwaters with low and high levels of plastic ingestion. For seabirds that ingest high quantities of plastic, the detection of intracellular proteins in the plasma suggests increased cell lysis, while the reduction in secreted proteins suggests multiple organ dysfunction.

### Multiorgan failure

The stomach is the initial site of ingested plastic where macroparticles often accumulate. The observed increase of stomach lining proteins detected within the plasma demonstrates a breakdown in the integrity of the stomach’s permeability. GKN2 assists in protecting and maintaining the stomach lining, while PGA5 is a precursor to pepsin, an enzyme that assists in the digestion of dietary proteins (*29*). PGA5 is only activated in low pH [1.5 to 2; (*40*)], which therefore has no function outside the stomach away from the acidic environment. The decrease in the stomach’s permeability is likely contributing to the translocation of small nanoplastic particles or plastic-derived chemical leachates. We have previously identified that the inflammation surrounding microparticles embedded within the stomach tissue is associated with the development of a fibrotic disease called plasticosis (*16*).

The proteomic signature observed for GPX3 supports previous findings that filtration organs such as the kidneys are affected through microplastic exposure (*13*). Other populations of wildlife have been brought to near extinction by renal toxic substances such as nonsteroidal anti-inflammatory drugs including diclofenac (*41*); therefore, it is concerning to see signals of renal toxicity in plastic-exposed seabird fledglings. Furthermore, plasma GPX3 is essential in mitigating reactive oxygen species damage, which can be induced by multiple stressors including physical exertion, infection, and inflammation. The reduced levels of this protective antioxidant enzyme before migration in the plastic-affected birds are limiting the normal physiological response we see in migratory birds where they up-regulate the antioxidant capacity before departure to mitigate the oxidative stress of long-distance flight (*42*, *43*).

**Fig. 5.**
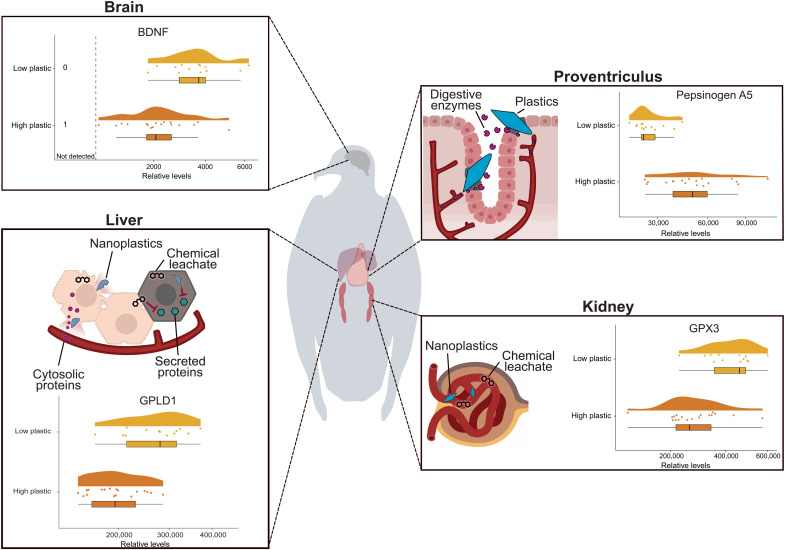
Plastic ingestion induces proteomic signatures distal to the site of exposure. Anatomical diagram of sable shearwater (*A. carneipes*) exposed to low (*n* = 13) and high (*n* = 18) quantities of ingested plastics. Proteomics on the plasma of chicks exposed to high quantities of ingested plastic revealed loss of brain, liver, and kidney function and stomach integrity disruption. Proteins of interest include BDNF, GPLD1 (glycosylphosphatidylinositol-specific phospholipase D1), GPX3, and PGA5.

Hypoalbuminemia (low levels of albumin) detected within the blood can be associated with liver failure, but as homeostasis of albumin requires only a partially functioning liver, this association typically occurs during chronic and very severe cases of liver failure (*44*). More commonly, hypoalbuminemia is associated with kidney failure and permeability of the gut, causing albumin to leak into the blood (*45*). This suggests that the hypoalbuminemia observed is a multifaceted condition in the seabird chicks heavily affected by plastic ingestion and is potentially induced by a combination of one or more of these three factors: liver damage, kidney damage, and/or gut permeability (Fig. 5).

In a medical context, decreases in relative levels of BDNF are a marker of neurological dysfunction and neuronal loss and are associated with neurodegenerative diseases, including Alzheimer’s, Huntington’s, and Parkinson’s disease (*46*). It has additionally been observed in several forms of dementia and associated with age-related cognitive decline (*34*). In birds, BDNF has been associated with the development of the song control system (*47*–*49*), suggesting that, while survival to adulthood is unlikely in plastic-affected birds, should they survive, there may be problems with the cognitive function needed for courtship. This will affect the breeding success of seemingly healthy birds. For shearwaters and other colonial breeding birds, fine-scale acoustic cues are essential for effective communication between lifelong pairs in large noisy nocturnal colonies (*50*, *51*). Male-female reunion requires identification of the call of the opposite sex and individual mate recognition (*52*). Interruption of the development of song control and the ability to discriminate sound could have considerable implications for fitness, particularly in long-lived species exposed to plastic.

Brain-specific proteomics further validated the findings from the plasma proteomics. While BDNF was not detected in the brain tissue proteomics likely due to the relative abundance in the samples, signatures of brain damage were observed. Key markers of neuroplasticity [microtubule-associated protein 1A (MAP1A) and growth-associated protein 43 (GAP43); (*35*, *36*)], fibrosis [PDGFRβ; (*38*)], and oxidative stress [SOD1; (*39*)] were up-regulated in the plastic-affected birds. These signatures have also been reported to be up-regulated in ischemic stroke (*36*), traumatic brain injury (*37*), and dementia (*53*). Similarly under these conditions, decreases in plasma BDNF are observed (*54*–*56*). While as little as 0.1 g of ingested plastic is enough to significantly enrich these pathways, the relative level of each marker is positively correlated with greater ingested plastic load (Fig. 4). The brain injury observed is likely due to the translocation of microplastics and loss of blood-brain barrier integrity (*39*, *57*). The observed enrichment of PDGFRβ supports this hypothesis as a primary function of this pathway is to support the repair of damaged blood vessels and promote angiogenesis, which assists in the recovery of the injured brain tissue (*58*). Second, PDGFRβ contributes to the development of fibrosis (*38*). Excessive PDGFRβ signaling can lead to extensive scarring, reducing the functional recovery of the brain following the injury, resulting in a loss of brain function (*38*).

### Proteomic differences because of gene regulation and cell lysis

There was significant enrichment of detoxification, antioxidant, and cellular stress proteins. Notable examples include alcohol dehydrogenase, aldehyde dehydrogenase, GPXs, heat shock proteins, peptidylprolyl isomerase A, and SOD1. These pathways are up-regulated following tissue damage (such as damage caused by translocated nanoplastics) and chemical exposure of plastic-derived leachates and/or additional chemicals that plastic may harbor (*59*). For example, Chen *et al.* (*60*) demonstrated that plastic exposure induces oxygen reactive species production and heat shock protein up-regulation in blood vessel cells. However, the increase in plasma concentration of these proteins may additionally be explained by cell lysis. The proteins are found intracellularly throughout the body, and so, tissue damage and cell lysis will also induce increased plasma concentrations. It is plausible that both the up-regulation of these pathways and increased cellular release explain the plasma signal observed here (*61*). The transcriptomic validation supports up-regulation of antioxidant pathways as *SOD1* gene expression was up-regulated in the liver and stomach of seabirds with high plastic ingestion rates (Fig. 3). Furthermore, the brain tissue proteomics revealed an increase in SOD1 in the brain. These combined signify an adaptive cellular response to an increase in oxidative stress.

### Using proteomics to reveal sublethal consequences of plastic exposure

Despite these birds appearing healthy, with no obvious signs of plastic ingestion (e.g., malnutrition), the proteome data suggest that the health of the birds is compromised. Typically, conservation biologists face many challenges when working with nonmodel organisms in investigating the health consequences and associated effects from contaminant exposure as a proteomic analysis is dependent on a suitable reference database of protein signatures for the organisms of interest. A potential limitation of this study is that fewer than 200 protein entries exist in the National Center for Biotechnology Information (NCBI) for *A. carneipes*. However, we were able to profile undepleted plasma to a depth of >700 proteins using the sequence database for the closely related species, *Fulmarus glacialis*, therefore overcoming this limitation. Many proteins are highly conserved across taxa, and there are many public databases available for related species and/or humans. This not only means that the outcomes observed from plastic ingestion in this study are comparable to a range of species exposed to plastic (including humans), but it also highlights the potential for research to be undertaken in nonmodel organisms. Future research could integrate metabolomics with proteomic methods, as metabolomics is not dependent on the organism being studied and can provide complementary evidence to support the pathways identified through proteomic analyses.

The proteomic signatures identified in this research concerningly correlate with clinical signatures of widespread organ damage. However, the mechanism by which plastics induce these effects is yet to be elucidated. Microplastic shedding is a strong possibility where microplastics migrate around the body and penetrate organs. Here, they may induce clotting (*62*), reactive oxygen species damage (*63*), and inflammation (*13*). In addition, leachates from plastics including additives, such as triphenyl phosphate, phthalates, and benzotriazole ultraviolet (UV) absorbers, have established detrimental effects on tissues (*64*–*66*). Environmental plastics adsorb inorganic and organic pollutants (*67*), and there are over 350,000 chemicals and mixtures registered for production and use, 50,000 of which are confidential and a further 70,000 of which have unknown composition (*68*). The diversity and complexity of environmental plastics and the lack of verified spectral reference libraries pose challenges when deciphering the mechanisms of actions and associated chemical toxicity. Future research should implement nontarget screen using high-resolution mass spectrometry to identify putative pathogenic leachates. Last, it has recently been reported that plastic ingestion alters the gut microbiome, leading to an increase in the presence of pathogenic bacteria (*69*). Collectively, these diverse pathogenic properties of plastics may be having synergistic detrimental effects on multiple organ systems and further research is needed to establish the individual contribution or mechanistic links of these on the observed biological health consequences. However, establishing these mechanisms of action will ultimately require a concerted effort across many research groups performing intervention exposure studies using cell and animal model systems.

### Summary

Revealing the proteomic changes in nonmodel organisms exposed to plastic pollution is critical in understanding the harm caused by this pervasive pollutant. This study has shed light on the complexity of sublethal health consequences that otherwise remain invisible. Metrics commonly used within ecology for assessing body condition may be an ineffective tool for accurately documenting the impacts of plastic on populations. This research has demonstrated that the impacts of plastic extend beyond the point of initial exposure (i.e., the stomach) and affect many vital organs. The observed proteomic signatures of cell lysis, multiorgan failure, and neurodegeneration in <90-day-old chicks clearly indicate that the health of these birds is substantially compromised. Data-driven approaches using medical research techniques and environmental monitoring fields have delivered notable, cost-effective, and timely discoveries [e.g., (*70*)], and the plastics crisis warrants an equivalent response.

## MATERIALS AND METHODS

### Experimental design

The primary objective is to investigate the physiological responses of sable shearwater *A. carneipes* chicks to exposure of low or high quantities of ingested plastic using DIA-MS. This experiment is performed on plasma collected from live seabird chicks of equivalent body size and condition to assess the impacts of plastic ingestion of a free-living nonmodel organism. Validation of results was performed through DIA-MS proteomic analysis of brain tissue and qPCR of stomach and liver samples from necropsied chicks that unsuccessfully fledged.

### Ethical statement

Animal handling and sampling conducted in 2023–2024 for proteomic analyses were approved by the Charles Sturt University Animal Ethics Committee (permit number A22382). Animal handling and sampling conducted in 2022 for transcriptomic analyses were approved by the University of Tasmania Animal Ethics Committee (permit number A18480). All field sampling was conducted under the approval of the New South Wales Office of Heritage (scientific license no. SL100619) and Lord Howe Island Board (permit number 07/18) for access and use of natural resources for scientific purposes. All work was completed in compliance with local and national ethical regulations.

Seabird fledglings of a healthy weight were briefly handled to obtain body morphometrics and lavaged for ingested plastic load, their blood samples were drawn, and they were rereleased to the environment. Unsuccessful postfledgling seabirds were collected as beach-washed individuals and were euthanized because of extremely low body condition (<400 g).

### Seabird ingested plastic and blood sampling for proteomic analyses

Sable shearwater chicks (80 to 90 days old) were captured by hand at night on the colony surface on Lord Howe Island, Australia (31.554°S, 159.085°E) between 26 April and 10 May 2023. For each bird, morphometric data were recorded including body mass (±10 g) using a spring balance, flattened and straightened wing chord (±1 mm) using a stopped ruler, and head + bill and culmen length (±0.1 mm) using Vernier calipers. Measurements were collected by the same, experienced team member (J.L.L.) to ensure consistency. All birds had a numbered, metal band applied to the leg for identification. An important feature of this study is that all chicks included in the study were deemed to be in good health (based on morphometrics), with no visible signs of disease.

Ingested plastic was collected by stomach flushing (lavage) following procedures outlined by Duffy and Jackson (*71*) and detailed by Lavers *et al.* (*72*). Following lavage, plastic pieces were dried, counted, and weighed to the nearest 0.0001 g. In recent years, the substantial and increasing quantity of plastic fed to some chicks (>250 pieces) by the parent birds means that the long-standing plastics removal and quantification method (i.e., lavage) is sometimes incomplete; not all items can be removed using lavage because of the volume of plastic creating a blockage. For the “plastic-affected” birds, ingested plastic is reported as greater than the number of items we managed to successfully remove.

**Fig. 6.**
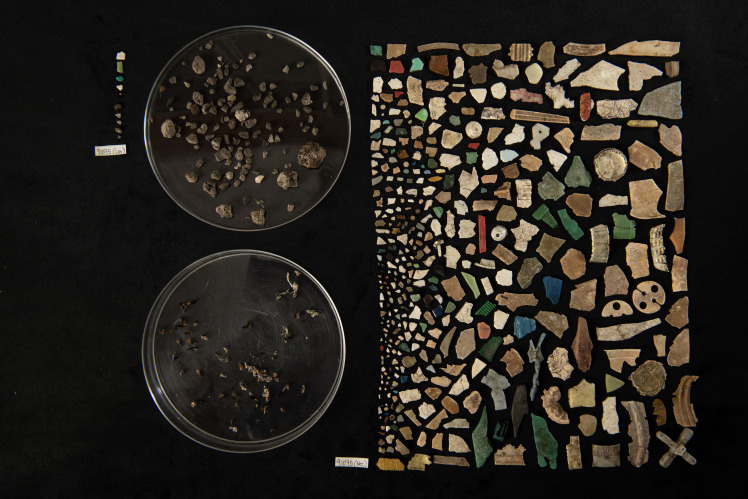
Four hundred three pieces of plastic removed from sable shearwater *A. carneipes* 90-day-old chick. On the left are five plastic items (0.073 g) removed during lavage, and on the right are the 398 items (41.812 g) that remained inside the stomach after lavage. The 398 items could not be removed during lavage as the large volume of plastic created a blockage. Instead, these items were recorded during necropsy after the bird was deceased. Pumice (128 pieces, 6.819 g) and squid beaks (not counted) also ingested by the bird are displayed in the petri dishes. The image was captured by J. Gilligan. This is the largest count record of plastic ingestion published for this species.

Chicks were allocated as “low” and “high” exposure categories if they met the following criteria: Bond and Lavers (*73*) found that the mass of plastic ingested cannot be used as a predictor of plastic count; therefore, birds were deemed as low plastic exposure if either <5 pieces or <0.5 g was obtained, and those with either higher mass or count were categorized as high plastic exposure. Highly plastic-exposed birds contained 8 to 403 pieces of ingested plastic weighing 0.65 to 41.89 g. One exception was made, where we were unable to remove all ingested plastic items using lavage techniques because of a blockage or impaction of plastic. From lavage, we removed four pieces of plastic weighing 0.484 g; however, it was readily apparent that a large volume of plastic remained within the seabird chick (e.g., Fig. 6) and the chick was allocated to the “high plastic” category.

Thirty-one blood samples were collected from chicks (18 high plastic and 13 low plastic). Approximately 1 ml of whole blood was collected from the brachial vein of each bird using a 26-gauge needle. In the field, samples were placed in Eppendorf tubes containing 50 μl of trisodium citrate to prevent inconsistent blood clots. Upon returning to the research station (~1 hour later), blood samples were centrifuged at 10,000 rpm for 5 min. Plasma was retained and stored at −20°C in cryotubes for the duration of the field trip (up to 13 days). All samples remained frozen during transportation and were then transferred to −80°C storage until laboratory processing.

### Protein extraction and digestion (plasma)

Plasma samples (20 μl) were diluted into 150 μl of T-PER Tissue Protein Extraction Reagent plus EDTA-free protease inhibitor cocktail (Roche, Basel, Switzerland). Protein samples were quantified by spectrophotometry (DeNovix) using the Pierce 660-nm Protein assay. Proteins samples (30 μg) were reduced and then alkylated using 10 mM dithiothreitol at 4°C overnight and 50 mM iodoacetamide for 2 hours at room temperature in a light-proof box, respectively. Proteins were then digested at a 1:25 enzyme:protein ratio with proteomics-grade trypsin/LysC (V5071; Promega, Madison, WI) overnight at 37°C according to the SP3 method (*74*). Following digestion, peptides were acidified with 10 μl of 1% trifluoroacetic acid and then desalted using C18 ZipTips (Millipore, cat. no. Z720070-96EA).

### Protein extraction (brain tissue)

Proteins were extracted from 20 mg of brain tissue using 7 M urea (Thermo Fisher Scientific), 2 M thiourea (Thermo Fisher Scientific), 10 mM BioUltra DI-dithiothreitol (Sigma-Aldrich), 30 mM tris-HCl (pH 8; Thermo Fisher Scientific), and EDTA-free protease inhibitor cocktail (Roche, Basel, Switzerland) digestion buffer. Protein samples were quantified by spectrophotometry using a NanoDrop ND-1000 UV-vis spectrophotometer (NanoDrop Technologies) by the Pierce 660-nm Protein Assay. Protein digestion followed that performed for the plasma samples defined above. The following experimental steps were as defined below and were consistent between plasma and brain samples, with the exception of the statistical test used. There was greater variation in results within the brain samples, and consequently, they were evaluated with linear models to determine dose-dependent results. Where necessary, data were Box-Cox transformed and tested for equal variance with residuals versus fitted plots.

### High-performance liquid chromatography and data-independent acquisition mass spectrometry

Peptide samples were analyzed by DIA-MS using an Ultimate nanoHPLC system coupled with a Q-Exactive HF mass spectrometer and controlled using Xcalibur software (version 4.4, Thermo Fisher Scientific). Instrument settings and the conditions used for nanoHPLC (nano–high-performance liquid chromatography) peptide separation are described elsewhere (*75*). Briefly, peptides equivalent to ~1 μg of each protein sample were separated using a 120-min gradient at a flow rate of 300 nl/min using a PepMap 100 C18 analytical column (250 mm by 75 μm) and were held at 45°C. Data acquisition parameters are as follows: 2.0-kV spray voltage, S-lens RF level of 60, and heated capillary set to 250°C. MS1 spectra (390 to 1240 *m/z*) were acquired in profile mode at a 120,000 resolution with an automatic gain control (AGC) target of 3 × 10^6^. The MS2 scans were acquired across 26 DIA–by–25 amu windows over the range of 397.5 to 1027.5 *m/z*, with a 1-amu overlap between windows.

### Protein identification, quantification, and normalization

DIA-MS raw files were processed using Spectronaut software (Biognosys), version 18 for plasma samples and version 19 for the brain samples. Project-specific peptide libraries for plasma and brain samples were generated using the Pulsar search engine to search the DIA MS2 spectra against the NCBI *F. glacialis* protein sequence database (TXID: 30455; composed of 28,615 entries, August 2023). Reextraction of MS1 and MS2 profiles was used for protein quantification and cross-run normalization according to Bionosys factory settings, with the exception that single-hit proteins were excluded. Spectronaut reports comprising protein label-free quantification (LFQ) intensity values were imported into R (version 4.2.2) for data filtering and statistical analysis.

### Proteomic bioinformatics and statistical analysis

Baseline statistics comparing low plastic-exposed birds with highly plastic-exposed birds were analyzed with general linear models for continuous variables. Where necessary, data were Box-Cox transformed for normality and Welch corrected to adjust for inequal variance. For count data, generalized linear models were performed with Poison distribution family. The appropriateness of all models was assessed with *Q*-*Q* plots and residuals versus fitted model. Baseline statistics were Holm-Šídák adjusted for multiple comparisons.

Many proteins were only identified in a few birds; to test for differences in the 822 proteins detected in the highly and low plastic-exposed groups, Fisher’s exact test was performed. Following this, the 745 proteins identified in more than 70% of samples in both groups (low or high plastic) were then evaluated with Mann-Whitney tests. Where necessary, data were Box-Cox transformed and tested for equal variance with residuals versus fitted plots. Fisher’s test and Mann-Whitney test *P* values were false discovery rate (FDR) adjusted, and *P* < 0.05 was deemed significant. A scaled PCA plot was generated to visualize the different clusters that best describe the maximum variance within the dataset.

As we are working with a nonmodel organism with proteomic data with unknown expected frequencies of the detect proteins, an adjusted enrichment analysis was performed. We first mapped the pathways of the proteins for the low plastic-exposed group using Gene ontology (GO), Kyoto Encyclopedia of Genes and Genomes (KEGG), and WikiPathways databases. Next, we mapped the pathways of the proteins which were significantly different between the highly and low plastic-exposed groups (FDR-corrected *P* value <0.05). A permutation test was then applied with 10,000 resampling events of the low plastic-exposed group data to evaluate the probability of the enrichment pattern observed in the significantly different protein dataset given random sampling.

Proteins found to be significantly different between highly and low plastic-exposed groups were also mapped to organ location and secretion or intracellular using the Human Protein Atlas (*76*) and Human Secretome (*77*) datasets. Genes of interest based on pathway enrichment and organ and secretion status were then visualized using heatmaps, raincloud plots, and volcano plots. All analyses were performed using R (version 4.2.2) (*78*) in RStudio (version 2023.3.0.386) (*79*); enrichment analyses were performed using enrichR (*80*); data visualization used pheatmap (*81*), ggplot2 (*82*), PupillometryR (*83*), sjPlot (*84*), and viridis (*85*) packages; the stringr (*86*) package was used for data handling; and the MASS (*87*) package was used for Box-Cox transformations. Data can be found in ProteomeXchange, and the code can be found in GitHub, with links provided below.

### Seabird ingested plastic and tissue procurement for validation of proteomic results

Sable shearwater chicks (80 to 90 days old) were captured by hand on Lord Howe Island, Australia (31.554°S, 159.085°E), between 26 April and 10 May 2022 and 2024. Birds were euthanized under permit after an unsuccessful fledgling attempt (*n* = 28) and/or because of low body mass (<400 g; *n* = 23) and were processed within 5 min of euthanasia. Chicks with pathologies identifiable during processing were noted and removed from analysis. Pathologies include skin lesions, poor feather condition, deformities, and extreme low body mass (<220 g).

In the field, liver and proventriculus samples of ~1 cm^3^ were dissected. Each tissue was rinsed in PBS and RNAlater and frozen at −20°C. Brains were removed from the whole skull, and the hippocampus was dissected and fresh frozen for proteomic analysis. All samples remained frozen during transportation and were then transferred to −80°C storage until laboratory processing. During necropsy, the ingested plastics in the proventriculus and gizzard of each chick were removed, dried, counted, and weighed to the nearest 0.001 g.

### RNA extraction from tissue and cDNA conversion

RNA was extracted from 20 mg of tissue using Promega ReliaPrep RNA Miniprep Systems with deoxyribonuclease I treatment following the manufacturer’s instructions (Promega Corporation, Madison, USA). Immediately following extraction, the RNA concentration and purity of each sample were assessed using a NanoDrop ND-1000 UV-vis spectrophotometer (NanoDrop Technologies). Samples with an RNA concentration <100 ng/μl with an *A*_260/280_ (absorbance at 260/280 nm) ratio of ~2.0 and *A*_260/230_ ratio between 2.0 and 2.2 were further processed (liver samples, *n* = 36; proventriculus samples, *n* = 13). Mucosal surfaces contain ribonucleases that catalyze the breakdown of RNA to make it difficult to obtain quality RNA out of stomach tissue (*88*).

The iScript cDNA Synthesis Kit (Bio-Rad Inc., Hercules, CA) was used for first-stand cDNA synthesis following the manufacturer’s protocol. Briefly, 1 μg of purified total RNA was used in a 20 μl reaction mixture containing reverse transcriptase, ribonuclease inhibitors, deoxynucleotide triphosphates (dNTPs), primers, MgCl_2_, and stabilizers. Samples were incubated at 25°C for 5 min, 46°C for 20 min, and 96°C for 1 min using the Veriti 96-well thermal cycler (Applied Biosystems). cDNA served as a template for reverse transcription qPCR.

### Gene expression analysis

cDNA was subjected to qPCR using the QuantStudio 3 Real-Time PCR system (Applied Biosystems) and PowerUp SYBR Green Master Mix (Applied Biosystems). A standard fast mode amplification program [95°C for 20 s; 40 cycles of 95°C for 1 s and 60°C for 20 s (data collected); followed by a melting curve analysis at 95°C for 1 s, 60°C for 20 s, and 95°C for 1 s (ramp rate, 0.1°C/s; data collected)] was used for all amplifications. Each reaction, including negatives, was performed in triplicates on a 96-well plate at a final PCR reaction volume of 10 μl. The results of qPCR runs were obtained using QuantStudio Design and Analysis (version 2.8.0; Applied Biosystems).

To validate the proteomic results, genes of interest were selected for quantification and included *ALB*, *GKN2*, and *SOD1*. As we are researching a nonmodel organism, primers (Table 3) were designed using NCBI Primer-BLAST (www.ncbi.nlm.nih.gov/tools/primer-blast/) and matched to Adélie penguin *Pygoscelis adeliae* (TaxId:9238) and northern fulmar *F. glacialis* (TaxId: 30455). Sequence alignment and primer conservation between the two species were confirmed using MEGA (version 11.3.13). Hydroxymethylbilane synthase (HMBS) was selected as a housekeeping gene as other commonly used housekeepers were found to be up-regulated in the proteomic results (i.e., GAPDH).

**Table 3.** Oligonucleotides used in this study.

Target gene	Primer name	Primer sequence (5′-3′)
Albumin	ALB_3_Forward	TGCAATGGCTGACTGCTGTA
Albumin	ALB_3_Reverse	GAACGAAGTCTGGCTGGGAA
Gastrokine-2	GKN2_1_Forward	TGAAGTCCATGTCCGTTCTGG
Gastrokine-2	GKN2_1_Reverse	TCTCTCAAAAGCCAGGCGTC
Hydroxymethylbilane synthase	HMBS_2_Forward	GTCAACGACCGGAGACAAGA
Hydroxymethylbilane synthase	HMBS_2_Reverse	CCGATGGTAAAGCCAGGAGG
Superoxide dismutase 1	SOD1_1_Forward	TGATGACCTGGGTAGAGGGG
Superoxide dismutase 1	SOD1_1_Reverse	ACACCACAAGCTAAACGAGGT

### Transcriptomic bioinformatics and statistical analysis

The expression of the genes of interest was normalized to the housekeeping gene using the delta-delta (ΔΔ*C*_t_) method. Where necessary, data were Box-Cox (*87*) transformed and tested for equal variance with *Q*-*Q* plots and residuals versus fitted plots. Appropriate transformations were applied when necessary. Gene expression differences between seabird chicks with low and high plastic ingestion rates were analyzed with general linear models. Statistical evaluations were deemed significant if *P* < 0.05. All analyses were conducted in R version 4.2.2 and RStudio 2023.03.0 + 386 (*79*).
