# Supplementary material for: Seabirds in crisis: Plastic ingestion induces proteomic signatures of multiorgan failure and neurodegeneration
Source: Sci Adv. 2025 Mar 12;11(11):eads0834. doi: 10.1126/sciadv.ads0834 (PMC11900854; doi:10.1126/sciadv.ads0834)
Supplement: Supplementary file 1 — Tables S1 to S3 [file sciadv.ads0834_sm.pdf]

Science Advances
AAAS

Supplementary Materials for

Seabirds in crisis: Plastic ingestion induces proteomic signatures of multiorgan failure and neurodegeneration

Alix M. de Jersey *et al.*

Corresponding author: Jack Rivers-Auty, jack.auty@utas.edu.au

Sci. Adv. **11**, eads0834 (2025)
DOI: 10.1126/sciadv.ads0834

This PDF file includes:

Tables S1 to S3

Table S1. Proteomics reveals that plastic ingestion causes enriched signatures of cell lysis, neurodegeneration, response to infection, and damage mitigation and repair. The 745 proteins identified through proteomics of Sable Shearwater (*Ardenna carneipes*) chicks exposed to both low (n = 13) and high (n = 18) quantities of ingested plastic were mapped to function, pathway or location using Gene Ontology (GO), Kyoto Encyclopedia of Genes and Genomes (KEGG), WikiPathway and Transcription Regulatory Relationships Unravelled Sentence-bases Text (TRRUST) databases. Proteins were evaluated for significance using Mann-Whitney tests, with FDR corrections applied. Those that significantly differed between the low and high plastic ingestion groups following FDR correction were then analyzed for function, pathway, or location enrichment using permutation tests with 10,000 resampling events. All significantly enriched outputs were found to have increased abundance in the high plastic ingestion group.

Database	Enriched pathway	Genes	Gene enrichment fraction (plastic, control)
GO Biological Process 2023	Positive Regulation Of NF-kappaB Transcription Factor Activity (GO:0051092)	PSMA6, MTPN, NPM1, TFRC, UBE2N, AKR1B1, PPIA, AGT	8/152 ***
	Glycolytic Process (GO:0006096)	GPI, LDHA, TPI1, PKM, PGK1, ALDOC, ENO1, ALDOB, GAPDH	9/29 ***
	Positive Regulation Of Protein Metabolic Process (GO:0051247)	NIBAN1, RAB1A, MTPN, VCP, HSP90AA1, NPM1, DDX3X, MSN, PLXNB2, EEF2, AGT	11/196 ***
	Pyruvate Metabolic Process (GO:0006090)	GPI, LDHB, LDHA, TPI1, PKM, PGK1, ALDOC, ENO1, ALDOB, GAPDH	10/50 ***
	Positive Regulation Of DNA-binding Transcription Factor Activity (GO:0051091)	PSMA6, MTPN, NPM1, TFRC, UBE2N, AKR1B1, PPIA, AGT	8/246 **
	Regulation Of Cell Differentiation (GO:0045595)	PPP2CA, PTBP1, BDNF, SERPINF2, AKR1B1, YWHAG	6/193 **
	Regulation Of Apoptotic Process (GO:0042981)	ITGB1, VCP, HSP90AA1, NPM1, DDX3X, TFRC, ACTN1, GLO1, ACTN4, HMGB1, YWHAZ, AGT, SOD1, COMP, OLFM1, ARHGDIA, ALB, PPIA, GPLD1, TPT1	20/705 **
	Positive Regulation Of Gene Expression (GO:0010628)	NIBAN1, NPM1, DDX3X, GSN, TFRC, MSN, AKR1B1, EEF2, AGT, ACTG1, SOD1, ACTA2, OLFM1, C1QTNF3, PLXNB2, PLA2R1, GAPDH	17/480 **
	Negative Regulation Of Intrinsic Apoptotic Signaling Pathway (GO:2001243)	DDX3X, ENO1, PPIA, TPT1	4/61 **
	Negative Regulation Of Apoptotic Signalling Pathway (GO:2001234)	DDX3X, GPX1, BDNF, AKR1B1	4/82 **
	Regulation Of RNA Metabolic Process (GO:0051252)	PTBP1, AHNAK, PCBP3, HNRNPH1, PRDX6	5/91 **

Database	Enriched pathway	Genes	Gene enrichment fraction (plastic, control)
	Cellular Response To Chemical Stress (GO:0062197)	DDX3X, GPX1, PPIA, SERPINB6	4/89 **
	DNA Metabolic Process (GO:0006259)	VCP, NPM1, UBE2N, HMGB3, HMGB1, RAN	6/288 **
	Regulation Of Autophagy (GO:0010506)	EEF1A1, PSAP, CAPN1, HMGB1, GAPDH	5/241 **
	Carbohydrate Catabolic Process (GO:0016052)	GPI, LDHA, TPI1, PKM, PGK1, ALDOC, ENO1, ALDOB, GAPDH	9/41 **
	Positive Regulation Of Translation (GO:0045727)	NIBAN1, NPM1, DDX3X, PKM, PLXNB2, PABPC1, EEF2	7/108 *
	Positive Regulation Of Macromolecule Biosynthetic Process (GO:0010557)	NIBAN1, MTPN, NPM1, DDX3X, PLXNB2, AKR1B1, EEF2	7/122 *
	Negative Regulation Of Apoptotic Process (GO:0043066)	ITGB1, NPM1, DDX3X, GPX1, TFRC, BDNF, GLO1, YWHAZ, SOD1, COMP, ARHGDIA, ALB, TPT1	13/482 *
	Regulation Of Translational Initiation (GO:0006446)	EIF4A2, NPM1, DDX3X	3/65 *
	Glycerophospholipid Catabolic Process (GO:0046475)	ENPP2, ENPP6, PRDX6	3/27 *
	Positive Regulation Of Developmental Process (GO:0051094)	TFRC, SERPINF2, MMRN2, ENPP2, AKR1B1	5/233 *
	Cellular Response To Superoxide (GO:0071451)	PRDX1, SOD3, SOD1	3/11 *
	Protein-Containing Complex Disassembly (GO:0032984)	HSPA8, VCP, LAMP2	3/62 *
	Regulation Of NLRP3 Inflammasome Complex Assembly (GO:1900225)	PPP2CA, HSPA8, DDX3X	3/33 *
	Regulation Of Macroautophagy (GO:0016241)	CAPN1, GAPDH, PAFAH1B2	3/96 *
	Regulation Of Programmed Cell Death (GO:0043067)	VCP, HSP90AA1, ASAH1, ACTN1, ALB, ACTN4, AGT, TPT1	8/195 *
	Superoxide Metabolic Process (GO:0006801)	PRDX1, SOD3, SOD1	3/34 *
	Autophagosome Maturation (GO:0097352)	VCP, LAMP2, CALM1	3/44 *
	Chromatin Remodeling (GO:0006338)	NPM1, GPX1, PSIP1	3/228 *
	Double-Strand Break Repair (GO:0006302)	VCP, UBE2N, HMGB1	3/168 *

Database	Enriched pathway	Genes	Gene enrichment fraction (plastic, control)
	Regulation Of Protein Localization To Nucleus (GO:1900180)	TFRC, F2, YWHAZ	3/52 *
	Regulation Of Viral Genome Replication (GO:0045069)	DDX3X, PABPC1, PPIA	3/67 *
	Positive Regulation Of Viral Genome Replication (GO:0045070)	DDX3X, PABPC1, PPIA	3/29 *
	Removal Of Superoxide Radicals (GO:0019430)	PRDX1, SOD3, SOD1	3/10 *
	Regulation Of Cation Channel Activity (GO:2001257)	ITGB1, AHNAK, CALM1	3/56 *
	Regulation Of Translation (GO:0006417)	EIF4A2, NIBAN1, MTPN, NPM1, DDX3X, PLXNB2, EEF2, GAPDH	8/202 *
	Regulation Of Endopeptidase Activity (GO:0052548)	PSMA3, SERPIND1, SERPINF1, SERPINF2, GAPDH, SERPINB6	6/69 *
	Positive Regulation Of Cellular Biosynthetic Process (GO:0031328)	NIBAN1, ADSL, HSP90AA1, NPM1, DDX3X, PLXNB2, EEF2, GPLD1	8/174 *
	Regulation Of Protein Catabolic Process (GO:0042176)	EEF1A1, VCP, HSP90AA1, GPX1, MSN	5/122 *
	Regulation Of Gene Expression (GO:0010468)	GPI, DDX3X, GPX1, GSN, TFRC, AHNAK, MSN, AKR1B1, PRDX6, HNRNPAB, ACTG1, ACTA2, PPP2CA, OLFM1, PTBP1, PCBP3, HNRNPH1	17/1127 *
	Positive Regulation Of Amide Metabolic Process (GO:0034250)	NIBAN1, NPM1, DDX3X, PLXNB2, EEF2	5/85 *
	Glucose Metabolic Process (GO:0006006)	GPI, TPI1, MDH1, PGK1, APOD, ENO1	6/59 *
	Positive Regulation Of Protein Catabolic Process (GO:0045732)	HSP90AA1, VCP, MSN, GPLD1	4/92 *
	Regulation Of MAPK Cascade (GO:0043408)	PEBP1, HMGB1, PPIA, YWHAZ, HLA-DRB1, SOD1	6/204 *
	Positive Regulation Of Protein Localization To Nucleus (GO:1900182)	CCT2, TFRC, F2, RAN	4/72 *
Go Cellular Component 2023	Nucleus (GO:0005634)	GPI, DDX3X, ADK, PEBP1, AKR1B1, PSIP1, ENO1, HMGB1, ACTG1, PTBP1, TUBA1C, TUBB6, CSRP1, HSP90AA1, TPI1, ACTN4, PGD, YWHAZ, SERPINB6, EEF1A1, PSMA6, PSMA3, PKM, HNRNPH1, EEF1D, CMPK1, MYH9, PABPC1, PPIA, GAPDH, KPNB1, CSTB, MTPN, VCP, AHNAK, PPP2CA, LDHA, CAND1, TUBA3C, PCBP3, PRDX1, PLTP, HSPA8, GSTM3, NPM1, GSN, HSPA4, MSN, ARPC5, EEF2, PRDX6,	61/4487 ***

Database	Enriched pathway	Genes	Gene enrichment fraction (plastic, control)
		HNRNPAB, LSM3, SOD1, ARPC2, ALB, UBE2N, S100P, CALM1, RAN, TPT1	
	Intracellular Membrane-Bounded Organelle (GO:0043231)	GPI, DDX3X, TFRC, ADK, PEBP1, AKR1B1, PSIP1, ENO1, HMGB1, ACTG1, PTBP1, TUBA1C, TUBB6, CSRP1, LAMP2, HSP90AA1, TPI1, ACTN4, PGD, YWHAZ, SERPINB6, EEF1A1, PSMA6, PSMA3, PKM, HNRNPH1, EEF1D, CMPK1, MYH9, DSG2, PABPC1, PPIA, GAPDH, GPLD1, KPNB1, CSTB, MTPN, VCP, AHNAK, PPP2CA, LDHA, CAND1, TUBA3C, PCBP3, PRDX1, PSAP, PLTP, HSPA8, GSTM3, NPM1, GSN, HSPA4, MSN, ARPC5, EEF2, PRDX6, HNRNPAB, LSM3, SOD1, ARPC2, ALB, UBE2N, S100P, CALM1, RAN, TPT1	66/5175 ***
	Cytoplasmic Vesicle Lumen (GO:0060205)	HSPA8, GPI, CSTB, VCP, HSP90AA1, DDX3X, GSN, ARPC5, HMGB1, EEF2, EEF1A1, PKM, CAND1, ALDOC, S100P, PPIA, VCL, PAFAH1B2	18/115 **
	Cytoskeleton (GO:0005856)	GSN, AHNAK, TPM2, DSTN, MSN, ACTN4, ARPC5, TUBA4A, ACTG1, PPP2CA, TUBA1C, TUBB6, TUBA3C, ARPC2, ARHGDIA, CAPZA2, MYH9, ALDOC, TAGLN3, PDLIM5, GAPDH, VCL, TUBA8	23/599 **
	Ficolin-1-Rich Granule Lumen (GO:1904813)	HSPA8, GPI, CSTB, VCP, HSP90AA1, ASAH1, DDX3X, GSN, ARPC5, HMGB1, EEF2, EEF1A1, PKM, CAND1, ALDOC, CAPN1, PPIA, KPNB1, VCL, PAFAH1B2	20/123 **
	Secretory Granule Lumen (GO:0034774)	GPI, CSTB, VCP, DDX3X, PROS1, PLG, HMGB1, CLEC3B, CAND1, TTR, AGA, HSPA8, CCT2, HSP90AA1, GSN, ACTN1, SERPINF2, ACTN4, ARPC5, EEF2, CYRIB, PRDX6, EEF1A1, PKM, SELENOP, ALB, ALDOC, S100P, PPIA, KPNB1, VCL, PAFAH1B2	32/316 **
	Microtubule (GO:0005874)	TUBA1C, CCT2, TUBB6, TUBA3C, DPYSL2, CALM1, TUBA4A, TUBA8	8/185 **
	Actin Cytoskeleton (GO:0015629)	GSN, WDR1, AHNAK, TPM2, ACTN1, DSTN, ACTN4, ARPC5, ACTG1, EEF1A1, ARPC2, CAPZA2, RAC2, PLS3, MYH9, TAGLN3, PDLIM5	17/327 **
	Axonal Growth Cone (GO:0044295)	OLFM1, HSP90AA1, FKBP4, NEO1	4/15 **
	Ficolin-1-Rich Granule (GO:0101002)	HSPA8, GPI, CSTB, VCP, HSP90AA1, ASAH1, DDX3X, GSN, ARPC5, HMGB1, EEF2, SERPINB6, EEF1A1, PKM, CAND1, LAMP2, ALDOC, CAPN1, PPIA, KPNB1, VCL, PAFAH1B2	22/184 **
	Microtubule Cytoskeleton (GO:0015630)	PPP2CA, TUBA1C, CCT2, TUBB6, TUBA3C, DPYSL2, GAPDH, TUBA4A, TUBA8	9/342 *

Database	Enriched pathway	Genes	Gene enrichment fraction (plastic, control)
	Cytoplasmic Stress Granule (GO:0010494)	VCP, DDX3X, PABPC1, KPNB1	4/76 *
	Polymeric Cytoskeletal Fiber (GO:0099513)	CCT2, TPM2, ACTN1, TUBA4A, ACTG1, TUBA1C, TUBB6, TUBA3C, DPYSL2, RAC2, PLS3, HLA-DRB1, TUBA8	13/265 *
	Vesicle (GO:0031982)	CBR1, TFRC, AHNAK, MSN, YWHAZ, TUBA1C, PKM, OLFML3, COL6A3, CALM1, PPIA, GAPDH, VCL	13/235 *
Go Molecular Function	RNA Binding (GO:0003723)	EIF4A2, CSTB, VCP, ARF1, DDX3X, TFRC, AHNAK, ADK, PEBP1, HMGB3, PSIP1, ENO1, HMGB1, PTBP1, PCBP3, CSRP1, PRDX1, YWHAG, HSPA8, HSP90AA1, NPM1, ACTN1, MSN, ACTN4, EEF2, YWHAZ, HNRNPAB, LSM3, EEF1A1, PSMA6, PKM, HNRNPH1, UBE2N, MYH9, PABPC1, FKBP4, PPIA, KPNB1, RAN, TPT1	40/1411 ***
	Ubiquitin Protein Ligase Binding (GO:0031625)	HSPA8, GPI, CCT2, VCP, HSP90AA1, PSMA3, TPI1, UBE2N, PRDX6, YWHAZ, VCL, ACTG1	12/271 ***
	Ubiquitin-Like Protein Ligase Binding (GO:0044389)	HSPA8, GPI, CCT2, VCP, HSP90AA1, PSMA3, TPI1, UBE2N, PRDX6, YWHAZ, VCL, ACTG1	12/289 ***
	Purine Ribonucleoside Triphosphate Binding (GO:0035639)	HSPA8, GPI, ARF1, HSP90AA1, HSPA4, TUBA4A, EEF1A1, RAB10, TUBA1C, PSMA6, TUBB6, TUBA3C, PGK1, RAC2, MYH9, RAN, TUBA8	17/476 ***
	Serine-Type Endopeptidase Inhibitor Activity (GO:0004867)	SPINT1, SERPIND1, SERPINC1, SERPINF1, SERPINF2, SERPINB6	6/53 **
	Cadherin Binding (GO:0045296)	ITGB1, HSPA8, RAB1A, DDX3X, AHNAK, ENO1, EEF2, PAICS, PRDX6, YWHAZ, RAB10, LDHA, ATIC, PKM, EEF1D, PRDX1, MYH9, S100P, PDLIM5, VCL, RAN	21/319 **
	GTP Binding (GO:0005525)	EEF1A1, RAB10, TUBA1C, TUBB6, ARF1, TUBA3C, RAC2, TUBA4A, RAN, TUBA8	10/201 *
	Oxidoreductase Activity, Acting On The CH-OH Group Of Donors, NAD Or NADP As Acceptor (GO:0016616)	CBR1, LDHB, LDHA, MDH1, PGD, ADH5	6/95 *
	Cell-Matrix Adhesion Mediator Activity (GO:0098634)	ITGB1, ITGA2, ITGA11	3/6 *
	Protein Heterodimerization Activity (GO:0046982)	ITGB1, PPP2CA, TPM2, PAFAH1B2, RAN	5/190 *
	Poly-Pyrimidine Tract Binding (GO:0008187)	PTBP1, HNRNPH1, PABPC1	3/25 *
	Guanyl Ribonucleotide Binding (GO:0032561)	EEF1A1, RAB10, TUBA1C, TUBB6, ARF1, TUBA3C, RAC2, TUBA4A, RAN, TUBA8	10/226 *

Database	Enriched pathway	Genes	Gene enrichment fraction (plastic, control)
	ATP Binding (GO:0005524)	HSPA8, GPI, HSP90AA1, HSPA4, PGK1, MYH9	6/279 *
	Protein Kinase Binding (GO:0019901)	ITGB1, HSP90AA1, NPM1, TFRC, PEBP1, MSN, AP2A1, EEF2, YWHAZ, TUBA4A, EEF1A1, ACTA2, CALM1, PDLIM5, YWHAG	15/511 *
	Kinase Binding (GO:0019900)	NPM1, GSN, TFRC, PEBP1, MSN, AP2A1, PLG, EEF2, YWHAZ, TUBA4A, EEF1A1, ACTA2, CALM1	13/460 *
	Protein Homodimerization Activity (GO:0042803)	GSTM3, HSP90AA1, NPM1, GSTM1, TPI1, TFRC, TPM2, ACTN1, SERPINF2, ENO1, ACTN4, ATIC, PSAP, MYH9, S100P, PAFAH1B2	16/662 *
	MHC Class II Protein Complex Binding (GO:0023026)	HSPA8, HSP90AA1, PKM, HLA-DRB1	4/25 *
KEGG 2021 Human	Tight junction	ITGB1, HSPA4, ACTN1, ARPC5L, MSN, ACTN4, ARPC5, ACTR3B, TUBA4A, ACTG1, PPP2CA, TUBA1C, TUBA3C, ARPC2, MYH9, TUBA8	16/169 ***
	Pathogenic Escherichia coli infection	ITGB1, RAB1A, ARF1, ARPC5L, ARPC5, F2, ACTR3B, TUBA4A, ACTG1, TUBA1C, TUBB6, TUBA3C, ARPC2, MYH9, GAPDH, TUBA8	16/197 ***
	Glycolysis / Gluconeogenesis	GPI, TPI1, ENO1, ADH5, MINPP1, LDHB, LDHA, PKM, PGK1, ALDOC, ALDOB, GAPDH, ALDH9A1	13/67 ***
	Huntington disease	TUBA1C, PSMA6, TUBB6, PSMA3, GPX1, TUBA3C, BDNF, GPX3, AP2A1, TUBA4A, TUBA8, SOD1	12/306 **
	Pathways of neurodegeneration	RAB1A, VCP, GPX1, BDNF, GPX3, TUBA4A, SOD1, TUBA1C, PSMA6, TUBB6, PSMA3, TUBA3C, CAPN1, CALM1, TUBA8	15/475 **
	Pyruvate metabolism	LDHB, LDHA, PKM, MDH1, GLO1, ADH5, ALDH9A1	7/47 **
	Regulation of actin cytoskeleton	ITGB1, GSN, ACTN1, ITGA2, ARPC5L, MSN, ACTN4, ARPC5, F2, ACTR3B, ACTG1, ARPC2, ITGA11, RAC2, MYH9, VCL	16/218 **
	Glutathione metabolism	GSTM3, GPX1, GSTM1, GPX3, PGD, PRDX6	6/57 **
	HIF-1 signaling pathway	LDHB, LDHA, TFRC, PGK1, ALDOC, ENO1, ALDOB, GAPDH	8/109 **
	RNA transport	EIF4A2, EEF1A1, PABPC1, KPNB1, RAN	5/186 **
	Phagosome	ITGB1, TFRC, ITGA2, THBS2, TUBA4A, ACTG1, COMP, TUBA1C, TUBB6, TUBA3C, LAMP2, PLA2R1, HLA-DRB1, TUBA8	14/152 *
	Gap junction	TUBA1C, TUBB6, TUBA3C, TUBA4A, TUBA8	5/88 *

Database	Enriched pathway	Genes	Gene enrichment fraction (plastic, control)
	Alzheimer disease	TUBA1C, PSMA6, TUBB6, PSMA3, TUBA3C, CAPN1, CALM1, GAPDH, TUBA4A, TUBA8	10/369 *
	Amyotrophic lateral sclerosis	RAB1A, VCP, GPX1, GPX3, TUBA4A, ACTG1, SOD1, TUBA1C, PSMA6, TUBB6, PSMA3, TUBA3C, TUBA8	13/364 *
	Legionellosis	EEF1A1, RAB1A, HSPA8, ARF1, VCP, SAR1B	6/57 *
	Salmonella infection	ARF1, HSP90AA1, AHNAK, ARPC5L, ARPC5, ACTR3B, TUBA4A, ACTG1, TUBA1C, TUBB6, TUBA3C, ARPC2, GAPDH, TUBA8	14/249 *
	Bacterial invasion of epithelial cells	ITGB1, ARPC2, ARPC5L, ARPC5, ACTR3B, VCL, ACTG1	7/77 *
	Endocytosis	RAB10, HSPA8, ARF1, TFRC, ARPC2, CAPZA2, ARPC5L, AP2A1, ARPC5, ACTR3B	10/252 *
	Hepatitis C	PPP2CA, YWHAQ, YWHAZ, YWHAG	4/157 *
	Drug metabolism	GSTM3, GSTM1, CMPK1, ADH5	4/108 *
	Metabolism of xenobiotics by cytochrome P450	CBR1, GSTM3, GSTM1, ADH5	4/76 *
	Necroptosis	HSP90AA1, CAPN1, HMGB1, PPIA	4/159 *
TRRUST Transcription Factors 2019	HSF1 human	LDHA, HSPA4, FKBP4	3/31 *
WikiPathway 2021 Human	Pathogenic Escherichia coli infection WP2272	ITGB1, TUBA1C, TUBB6, TUBA3C, YWHAQ, ARPC2, ARPC5L, ARPC5, YWHAZ, TUBA4A, ACTG1, TUBA8	12/55 ***
	Computational Model of Aerobic Glycolysis WP4629	GPI, LDHA, TPI1, PKM, PGK1, ENO1, GAPDH	7/12 ***
	Glycolysis and Gluconeogenesis WP534	GPI, LDHB, LDHA, TPI1, PKM, MDH1, PGK1, ALDOC, ENO1, ALDOB, GAPDH	11/45 ***
	Metabolic reprogramming in colon cancer WP4290	GPI, LDHA, PKM, PGK1, ENO1, ALDOB, PGD, TKT, PAICS, GAPDH	10/42 ***
	Glycolysis in senescence WP5049	LDHA, PKM, PGK1, ALDOC, ENO1, GAPDH	6/11 ***
	Nuclear Receptors Meta-Pathway WP2882	GSTM3, CBR1, HSP90AA1, SEC14L1, GSTM1, GPX3, PRDX1, S100P, PGD, PLTP, PRDX6, SOD3	12/319 **
	Translation Factors WP107	EIF4A2, EEF1A1, EEF1D, PABPC1, EEF2	5/52 **
	Parkin-Ubiquitin Proteasomal System pathway WP2359	TUBA1C, HSPA8, TUBB6, TUBA3C, HSPA4, TUBA4A, TUBA8	7/70 **

Database	Enriched pathway	Genes	Gene enrichment fraction (plastic, control)
	NRF2 pathway WP2884	GSTM3, CBR1, HSP90AA1, GSTM1, GPX3, PRDX1, PGD, PRDX6, SOD3	9/146 **
	Pathways in clear cell renal cell carcinoma WP4018	GPI, LDHB, LDHA, TPI1, PKM, MDH1, PGK1, ALDOC, ENO1, ALDOB, GAPDH	11/85 **
	VEGFA-VEGFR2 Signaling Pathway WP3888	ITGB1, HSP90AA1, GPX1, ARPC5L, AP2A1, HMGB1, PGD, PRDX6, ACTG1, PPP2CA, TUBA1C, LDHA, CSRP1, ALB, MMRN2, PGK1, MYH9, PABPC1, TKT, ACP1, GAPDH, VCL	22/432 *
	Vitamin B12 metabolism WP1533	F7, ALB, PLG, MAT1A, F2, SOD3, SOD1	7/50 *
	Selenium Micronutrient Network WP15	F7, GPX1, GPX3, PRDX1, SELENOP, ALB, PLG, F2, SOD3, SOD1	10/89 *
	Sudden Infant Death Syndrome (SIDS) Susceptibility Pathways WP706	YWHAQ, BDNF, YWHAZ, GAPDH, YWHAG	5/158 *
	HIF1A and PPARG regulation of glycolysis WP2456	LDHA, TPI1, GAPDH	3/8 *
	Glucocorticoid Receptor Pathway WP2880	HSP90AA1, SEC14L1, S100P	3/70 *
	Sterol regulatory element-binding proteins (SREBP) signaling WP1982	RBP4, MDH1, SAR1B, KPNB1	4/69 *
	One-carbon metabolism and related pathways WP3940	BHMT, GPX1, GPX3, MAT1A, SOD3, SOD1	6/52 *
	Regulation of Actin Cytoskeleton WP51	GSN, ACTN1, RAC2, MSN, ARPC5, F2, VCL, ACTG1	8/150 *
	Arrhythmogenic Right Ventricular Cardiomyopathy WP2118	ITGB1, ITGA2, ACTN1, ITGA11, DSG2, ACTN4, ACTG1	7/74 *
	Cori Cycle WP1946	GPI, LDHA, TPI1, PGK1, GAPDH	5/17 *

* P < 0.05, ** P < 0.01, *** P < 0.001.

Table S2. Proteomics on hippocampal sbrain tissue reveals that plastic ingestion causes enriched signatures of neuroplasticity. The 4,429 proteins identified through proteomics of Sable Shearwater (*Ardenna carneipes*) chicks exposed to both low (n = 5) and high (n = 5) quantities of ingested plastic were mapped to function, pathway or location using Gene Ontology (GO), Kyoto Encyclopedia of Genes and Genomes (KEGG), WikiPathway and Transcription Regulatory Relationships Unravelled Sentence-bases Text (TRRUST) databases. Proteins that significantly differed between the low and high plastic ingestion groups following FDR correction were then analyzed for function, pathway, or location enrichment using permutation tests with 10,000 resampling events. All significantly enriched outputs were found to have increased abundance in the high plastic ingestion group.

**Database**	**Enriched pathway**	**Genes**	**Gene enrichment fraction (plastic, control)**
GO Biological Process 2023	Regulation Of Phosphoprotein Phosphatase Activity (GO:0043666)	PDGFRB, NUAK1, PPP1R12A, GNA12, FKBP1B, HTT	6/37 ***
	Intermediate Filament Organization (GO:0045109)	KRT19, KRT4, DNAJB6, KRT15, KRT5, KRT6B	6/68 ***
	Lipid Transport (GO:0006869)	ACSL1, ATP8B1, FABP6, PITPNB, ABCA3, OSBPL2	6/108 **
	Phospholipid Transport (GO:0015914)	ATP8B1, PITPNB, ABCA3, OSBPL2, ATP11A	5/59 **
	Regulation Of Hydrolase Activity (GO:0051336)	FGD3, SIPA1L1, RDX, PLXNB2, OCRL, ARHGAP15, RAPGEF6, SOD1	8/71 **
	Plasma Membrane Bounded Cell Projection Morphogenesis (GO:0120039)	CNTNAP2, GPM6A, PAK1, SNX1, DICER1, SRGAP3	6/50 **
	Plasma Membrane Bounded Cell Projection Organization (GO:0120036)	GPM6A, CARMIL1, CNTNAP2, PLEKHA1, RASGRF1, TBC1D23, MAP4, PPP1R9A, MANF	9/132 **
	Regulation Of Calcium Ion Transmembrane Transporter Activity (GO:1901019)	FKBP1B, DMD, ANK2, JPH1	4/32 **
	Supramolecular Fiber Organization (GO:0097435)	CARMIL1, KRT4, TPM3, KRT5, PPP1R9A, KRT19, BIN3, DNAJB6, RUFY3, KRT15, MAP4, TARDBP, KRT6B	13/316 **
	Epidermis Development (GO:0008544)	HDAC2, KRT15, KRT5, ATP2C1	4/85 **
	Neuron Development (GO:0048666)	GPM6A, CNTNAP2, ARL3, RASGRF1, DMD, TBC1D23, MAP4, PPP1R9A, MANF	9/150 **
	Regulation Of Ras Protein Signal Transduction (GO:0046578)	SYNGAP1, DENND1A, MAPKAP1, RASGRF1, RDX, SHOC2	6/76 **
	Central Nervous System Development (GO:0007417)	CNTNAP2, CNTN5, ROGDI, ADAM22, EML1, PTS, SCIN, DCX, RAB18, PLXNB2, SPOCK1, TBC1D23, BPTF	13/283 **
	Generation Of Neurons (GO:0048699)	NRBP2, GPM6A, FZD3, NLGN4X, DCX, PLXNB2, SPOCK1, EML1	8/172 **

Database	Enriched pathway	Genes	Gene enrichment fraction (plastic, control)
	Positive Regulation Of Protein Catabolic Process (GO:0045732)	HSP90AA1, CSNK2A1, RDX, PACSIN3, MSN, SNX9	6/92 *
	Regulation Of GTPase Activity (GO:0043087)	RDX, RASGRF1, OCRL, ARHGAP15, SOD1, FGD3, SIPA1L1, PLXNB2, SNX9, SMCR8, SRGAP3, RAPGEF6, EVI5	13/214 *
	Lamellipodium Assembly (GO:0030032)	CARMIL1, GOLPH3, PTPRO, CDH13, SRGAP3	5/25 *
	Positive Regulation Of Actin Nucleation (GO:0051127)	SCIN, WASF2, WASF3	3/10 *
	Negative Regulation Of Cellular Component Organization (GO:0051129)	DNAJB2, CARMIL1, DNAJB6, HSPA5, RDX, PACSIN3	6/79 *
	Regulation Of Neuron Projection Development (GO:0010975)	PRKCI, HDAC2, PAK1, SIPA1L1, RUFY3, PTPRO, NPTN, PLXNB2, SPOCK1, KIF1A, FKBP4, PAK3	12/174 *
	Establishment Of Protein Localization To Membrane (GO:0090150)	PRKCI, PIKFYVE, GORASP2, RAB3IP, RDX	5/69 *
	Retrograde Transport, Endosome To Golgi (GO:0042147)	GGA1, SNX1, PIKFYVE, VPS53, SNX12, TBC1D23, EVI5	7/94 *
	Regulation Of Cation Channel Activity (GO:2001257)	FKBP1B, ANK2, KCNAB1, DMD, JPH1	5/56 *
	Plasma Membrane Bounded Cell Projection Assembly (GO:0120031)	FGD3, CARMIL1, GOLPH3, ARL3, RAB3IP, PTPRO, CDH13, OCRL, SRGAP3, FHDC1, RAPGEF6	11/275 *
	Inositol Lipid-Mediated Signaling (GO:0048017)	PDGFRB, OCRL, PI4KB, PLCB2	4/33 *
	Establishment Of Protein Localization To Plasma Membrane (GO:0061951)	GGA1, GOLPH3, GORASP2, RDX	4/38 *
	Phosphatidylinositol Metabolic Process (GO:0046488)	PDGFRB, FIG4, PIKFYVE, OCRL, PI4KB, PLCB2	6/63 *
	Cellular Response To Glucose Stimulus (GO:0071333)	UNC13B, PTPRN2, RAB11FIP5	3/34 *
	Regulation Of Protein Localization (GO:0032880)	VCPIP1, HSP90AA1, DNAJB6, MAP1A, TRIM25, KRT5, RAB11FIP5	7/97 *
	Plasma Membrane Tubulation (GO:0097320)	BIN3, PACSIN3, SNX9	3/13 *
	Positive Regulation Of Phosphoprotein Phosphatase Activity (GO:0032516)	PDGFRB, PPP1R12A, GNA12	3/18 *
	Regulation Of Calcium Ion Transport (GO:0051924)	WFS1, CACNA2D1, PACSIN3, ANK2	4/57 *
	Positive Regulation Of Protein Dephosphorylation (GO:0035307)	PDGFRB, PTBP1, GNA12, PPP2R5A	4/33 *

Database	Enriched pathway	Genes	Gene enrichment fraction (plastic, control)
	Lamellipodium Organization (GO:0097581)	CARMIL1, SNX1, GOLPH3, PTPRO, CDH13	5/30 *
	Neuron Projection Development (GO:0031175)	GPM6A, CNTNAP2, PAK1, RASGRF1, DICER1, TBC1D23, MAP4, SRGAP3, PPP1R9A, PAK3, MANF	11/192 *
	Vesicle Transport Along Microtubule (GO:0047496)	HTT, KIF1A, DYNC1I1	3/22 *
	Regulation Of Ryanodine-Sensitive Calcium-Release Channel Activity (GO:0060314)	FKBP1B, DMD, JPH1	3/24 *
	Retrograde Axonal Transport (GO:0008090)	MAP1A, KIF1A, SOD1	3/15 *
	Organic Hydroxy Compound Biosynthetic Process (GO:1901617)	OSBPL9, OSBPL2, PRKAG2, OSBPL1A	4/42 *
	Positive Regulation Of Cell Motility (GO:2000147)	PDGFRB, CARMIL1, PAK1, UBE2I, HSPA5, RUFY3, CCDC25, RDX, CDH13	9/221 *
	Regulation Of TOR Signaling (GO:0032006)	NUAK1, GOLPH3, SEH1L, GNA12, SMCR8	5/78 *
	Neuron Differentiation (GO:0030182)	NRBP2, FZD3, NLGN4X, SPOCK1, DMD, MANF	6/173 *
	Regulation Of Release Of Sequestered Calcium Ion Into Cytosol (GO:0051279)	FKBP1B, ANK2, DMD, JPH1	4/51 *
	Cardiac Muscle Cell Action Potential (GO:0086001)	CACNA2D1, DMD, SLC4A3, SCN1A	4/29 *
	Regulation Of Release Of Sequestered Calcium Ion Into Cytosol By Sarcoplasmic Reticulum (GO:0010880)	FKBP1B, ANK2, DMD	3/22 *
	Negative Regulation Of Cell Adhesion (GO:0007162)	PTPRO, ADAM22, CDH13, SPOCK1, PLXNB2	5/72 *
	Phosphatidylinositol-Mediated Signaling (GO:0048015)	PDGFRB, PLEKHA1, OCRL, PI4KB, PLCB2	5/62 *
	Endosomal Transport (GO:0016197)	GGA1, SNX1, PIKFYVE, DENND1A, VPS53, SNX12, SNX9, TBC1D23, VAMP4, EVI5	10/180 *
	Memory (GO:0007613)	ATAD1, RASGRF1, MAP1A, CPEB3	4/58 *
	Golgi Organization (GO:0007030)	VCPIP1, GOLPH3, SEC23IP, ATP8B1, GORASP2, HTT, FHDC1, CLASP2	8/130 *
	Cytoskeleton Organization (GO:0007010)	FGD3, PRKCI, KRT4, PALLD, MAPKAP1, PACSIN3, WASF3	7/111 *

Database	Enriched pathway	Genes	Gene enrichment fraction (plastic, control)
	Establishment Of Endothelial Barrier (GO:0061028)	RDX, MSN, RAPGEF6	3/28 *
	Cardiac Conduction (GO:0061337)	CACNA2D1, ANK2, SLC4A3, KCNJ3	4/46 *
	Brain Development (GO:0007420)	CNTNAP2, CNTN5, ROGDI, RAB18, PLXNB2, TBC1D23, EML1, BPTF	8/169 *
	Negative Regulation Of Neuron Projection Development (GO:0010977)	HDAC2, RUFY3, PTPRO, SPOCK1, FKBP4	5/54 *
	Negative Regulation Of Gene Expression (GO:0010629)	PTBP1, TIA1, CSNK2A1, CPNE1, SNX12, ERP29, NPTN, SMCR8, DICER1, CPEB3, TARDBP, EIF4E2	12/336 *
	Steroid Biosynthetic Process (GO:0006694)	OSBPL9, OSBPL2, PRKAG2, OSBPL1A	4/50 *
	Establishment Of Epithelial Cell Polarity (GO:0090162)	CYTH3, GOLPH3, MSN	3/19 *
	Positive Regulation Of MAP Kinase Activity (GO:0043406)	PDGFRB, PAK1, ERP29, MAP3K13	4/91 *
	Regulation Of Kinase Activity (GO:0043549)	PDGFRB, RAD50, SMG8, SOD1	4/85 *
	Homophilic Cell Adhesion Via Plasma Membrane Adhesion Molecules (GO:0007156)	PALLD, NPTN, CDH13, PLXNB2	4/60 *
	Negative Regulation Of Cysteine-Type Endopeptidase Activity Involved In Apoptotic Process (GO:0043154)	CSNK2A1, DNAJB6, DHCR24	3/49 *
	Negative Regulation Of Cysteine-Type Endopeptidase Activity (GO:2000117)	CSNK2A1, DNAJB6, DHCR24	3/53 *
Go Cellular Component 2023	Mitochondrial Ribosome (GO:0005761)	MRPL51, MRPL9, MRPL34	3/22 **
	Keratin Filament (GO:0045095)	KRT4, KRT5, KRT6B	3/39 **
	Early Endosome Membrane (GO:0031901)	GRIA1, FIG4, GGA1, SNX1, PIKFYVE, SNX12, OCRL	7/116 *
	Filopodium (GO:0030175)	GPM6A, GAP43, RUFY3, RDX, MSN, DMD	6/60 *
	Actin Cytoskeleton (GO:0015629)	PPP1R12A, TPM3, RDX, ARPC1A, LSP1, FHDC1, PPP1R9A, EEF1A1, PAK1, DIAPH2, SIPA1L1, SCIN, PALLD, ZYX, WASF2, DGKH	16/327 *
	Golgi Membrane (GO:0000139)	FIG4, CYTH3, PIKFYVE, ST8SIA1, GORASP2, PITPNB, GOLIM4, PI4KB, VPS45, VAMP4, ATP2C1, HS6ST2	12/427 *

Database	Enriched pathway	Genes	Gene enrichment fraction (plastic, control)
	Bounding Membrane Of Organelle (GO:0098588)	GRIA1, GRIA2, PTPRN2, ST8SIA1, PITPNB, GOLIM4, SLC2A3, ATP2C1, AP2A2, HS6ST2, FIG4, CYTH3, GGA1, SNX1, PIKFYVE, LAMP1, GORASP2, FKBP1B, RAB18, TAB3, PI4KB, VPS45, VAMP4	23/819 *
	Cytoplasmic Vesicle Membrane (GO:0030659)	GRIA1, GRIA2, PTPRN2, ABCA3, HTT, SLC2A3, AP2A2, FIG4, GGA1, SNX1, PIKFYVE, LAMP1, RAB18, TAB3, SNX9, VPS45	16/389 *
	Polymeric Cytoskeletal Fiber (GO:0099513)	KRT4, TPM3, KRT5, FHDC1, EML1, PAK1, DIAPH2, PALLD, MAP1A, KIF21A, MAP4, KIF1A, CLASP2	13/265 *
	Voltage-Gated Potassium Channel Complex (GO:0008076)	CNTNAP2, KCNQ2, KCNAB1, KCNJ3	4/73 *
	Actin Filament (GO:0005884)	PAK1, DIAPH2, TPM3, PALLD, FHDC1	5/71 *
	Dendrite (GO:0030425)	GRIA1, GRIA2, CNTNAP2, NLGN4X, WFS1, HTT, PPP1R9A, SOD1, PAK1, RUFY3, MAP1A, KIF1A, CPEB3, ZC3H14	14/270 *
	Excitatory Synapse (GO:0060076)	GRIA2, NLGN4X, PALLD	3/24 *
	Potassium Channel Complex (GO:0034705)	CNTNAP2, KCNQ2, KCNAB1, KCNJ3	4/81 *
	Ficolin-1-Rich Granule Membrane (GO:0101003)	PTPRN2, LAMP1, SLC2A3, AP2A2	4/60 *
	Tight Junction (GO:0070160)	CYTH3, PRKCI, AOC1, ILDR2, JAM2	5/93 *
	Axon (GO:0030424)	CNTNAP2, PAK1, CNTN5, PALLD, RUFY3, MAP1A, PTPRO, ADAM22, HTT, MAP4, KIF1A, ZC3H14	12/205 *
Go Molecular Function 2023	Microtubule Binding (GO:0008017)	ARL3, FHDC1, EML1, EML5, EML6, MAP1A, DCX, CCDC181, KIF21A, MAP4, KIF1A, DYNC1I1, CLASP2	13/239 **
	Tubulin Binding (GO:0015631)	ARL3, HTT, FHDC1, EML1, EML5, EML6, PAK1, MAP1A, DCX, CCDC181, KIF21A, MAP4, KIF1A, DYNC1I1, RAB11FIP5, CLASP2	16/322 **
	Phosphatidylcholine Transporter Activity (GO:0008525)	ATP8B1, ABCA3, PITPNB	3/17 *
	Protein Kinase A Regulatory Subunit Binding (GO:0034237)	AKAP7, WASF2, WASF3	3/22 *
	1-Phosphatidylinositol Binding (GO:0005545)	SCIN, SNX9, ZFYVE1	3/14 *
	Kinase Binding (GO:0019900)	PDGFRB, PPP1R12A, MAPKAP1, PRKAG2, HTT, MSN, PPP2R5A, ANK2, AP2A2, EEF1A1, MOB1B, FRMD5, PFKL, CDC37, DCX, CSPG4, SMCR8, MAP3K13	18/460 *

Database	Enriched pathway	Genes	Gene enrichment fraction (plastic, control)
	Phosphatidylinositol-3,4-Bisphosphate Binding (GO:0043325)	PLEKHA1, MAPKAP1, ZFYVE1	3/25 *
	Phosphatidylinositol Phosphate Binding (GO:1901981)	CYTH3, GAP43, GOLPH3, MAPKAP1, DENND1A, SNX12, ZFYVE1	7/109 *
	Phosphatidylinositol Binding (GO:0035091)	SNX1, GAP43, SCIN, PITPNB, DENND1A, SNX12, SNX9, ZFYVE1	8/106 *
	NF-kappaB Binding (GO:0051059)	HDAC2, CPNE1, COMMD7	3/26 *
	3'-5' Exonuclease Activity (GO:0008408)	PAN2, RAD50, ANGEL2	3/42 *
KEGG 2021 Human	Pentose phosphate pathway	PRPS2, TKTL1, PFKL, TKT, PFKP	5/30 **
	NF-kappa B signaling pathway	UBE2I, CSNK2A1, TRIM25, TAB3, PRKCQ	5/104 **
	Regulation of actin cytoskeleton	PDGFRB, PPP1R12A, RDX, ARPC1A, MSN, FGD3, PAK1, DIAPH2, PIKFYVE, SCIN, GNA12, PAK3, WASF2	13/218 *
	Protein processing in endoplasmic reticulum	DNAJB2, ERO1B, HSP90AA1, HSPA5, WFS1, HSPA4L, ERP29, CUL1, SEC61B, PDIA4	10/171 *

* P < 0.05, ** P < 0.01, *** P < 0.001.

Table S3. Comparisons of morphometric data of Sable Shearwater *Ardenna carneipes* chicks exposed to low (n = 5) and high (n = 5) quantities of ingested plastic sampled for brain proteomics. Count data is displayed as median with interquartile ranges (IQR). Continuous data is displayed as mean ± standard deviation (SD). Poisson generalized linear models for count data, and general linear models were used for continuous variables (corrections or transformations were applied where necessary). Comparisons were adjusted using Holm-Šídák post hoc corrections.

	**Low plastic**	**High plastic**	**Statistical test**
Weight (grams)	319 ± 69	298 ± 55	$t_{7.6} = 0.53$, NS
Wing length (mm)	269 ± 28	267 ± 28	$t_{8.0} = 0.07$, NS
Culmen length (mm)	51.2 ± 22.4	40.8 ± 1.3	$t_{4.0} = 1.04$, NS
Head + bill length (mm)	81.8 ± 23.9	92.9 ± 2.8	$t_{4.1} = 1.03$, NS
Ingested plastic count	3 (IQR 1 - 4)	26 (IQR 14 - 35)	$x^2_1 = 153.88$ ***
Ingested plastic mass (grams)	0.03 ± 0.02	2.03 ± 2.27	$R^2 = 0.746$, $F_{1, 8} = 23.52$, ***

NS not significant, *** $P < 0.001$.
